# Passive Smart Home Monitoring for Delirium-Relevant Anomaly Detection in People Living With Dementia: Proof-of-Concept Study

**DOI:** 10.2196/93258

**Published:** 2026-06-12

**Authors:** Cong Mou, Mian Wu, Shreyank N Gowda, Beili Shao

**Affiliations:** 1 School of Psychology Faculty of Science University of Nottingham Nottingham, England United Kingdom; 2 School of Computer Science Falculty of Science University of Nottingham Nottingham, England United Kingdom; 3 Academic Neurology, Academic Unit of Mental Health and Clinical Neurosciences School of Medicine University of Nottingham Nottingham, England United Kingdom; 4 Academic Neurology Queen’s Medical Centre University of Nottingham Nottingham United Kingdom

**Keywords:** delirium risk detection, smart home monitoring, dementia, anomaly detection, passive sensing

## Abstract

**Background:**

Delirium superimposed on dementia is associated with poor outcomes yet remains underdetected in home settings. Current detection relies on face-to-face clinical assessment (eg, the Confusion Assessment Method criteria), which is rarely applied outside hospitals.

**Objective:**

This proof-of-concept study developed a theory-driven framework for detecting delirium-consistent anomalous patterns in home-dwelling people with dementia, using passive smart home sensor data.

**Methods:**

The Technology Integrated Health Management dataset, an open access resource comprising a clinically derived cohort of older adults (aged 50 years) with a confirmed diagnosis of dementia or mild cognitive impairment, was used. The analysis included 13 patients who had at least 50% valid data for at least one 10-day analysis window, with data collected between April 1, 2019, and June 30, 2019. Individualized anomaly detection algorithms, including Isolation Forest and Long Short-Term Memory models, were applied to identify delirium-related anomalies within each participant. Predictor features consisted of theory-driven digital markers approximating key Confusion Assessment Method criteria, including agitation, disrupted sleep-wake cycles, and disorientation (indexed by activity entropy), along with clinically relevant indicators, such as physiological instability (early warning scores) and urinary tract infections.

**Results:**

Using matched thresholds, the Isolation Forest identified 77 anomalies (anomaly rate: 15.65%), and the Long Short-Term Memory model identified 78 anomalies (anomaly rate: 15.85%), with anomalies typically occurring in short temporal clusters; agreement between methods ranged from 0% to 40% across individuals. Feature importance analyses indicated that activity entropy, sleep quality, and early warning scores were the most influential features, with stronger interfeature correlations observed during anomaly periods than during nonanomaly periods.

**Conclusions:**

This study demonstrates the technical feasibility of detecting delirium-related anomalies through passive smart home monitoring. While lacking ground truth validation, the approach shows promise for early intervention in community settings. Future validation studies with clinically confirmed delirium labels are essential.

## Introduction

Delirium is a clinically diagnosed neuropsychiatric syndrome characterized by acute and fluctuating disturbances in attention, cognition, and arousal [1]. It is highly prevalent among people living with dementia (22%-89%, depending on setting) [2] and is associated with substantial burden, including distress for patients and caregivers, accelerated cognitive decline, increased institutionalization, and elevated mortality [3,4]. Despite these consequences, delirium remains underdetected in home and care home settings, particularly in its early stages when intervention may be most effective [5,6].

Early detection of delirium, particularly during the prodromal phase, offers a critical window for intervention. Community-based management is feasible and can prevent hospitalization in many cases [7]. When identified early in home settings, interventions can target precipitating factors (eg, infections, medications, and dehydration), manage emerging symptoms, and support recovery [8]. However, these benefits depend on timely and reliable detection methods, which remain limited outside clinical settings.

Despite the importance of early identification, current delirium detection relies heavily on clinical evaluation using tools, such as the Confusion Assessment Method (CAM) [9], which require face-to-face assessment by trained clinicians and are rarely applied outside acute care settings. Several challenges compound the difficulty of detecting delirium in people with dementia. First, cognitive tests commonly used to identify delirium in older adults are often confounded by preexisting dementia, reducing their diagnostic accuracy [10]. Second, many delirium symptoms rely on self-report, which becomes nearly impossible for individuals with advanced dementia [11]. Third, family caregivers often lack knowledge about delirium and its differentiation from medication side effects [5], further delaying recognition. These detection barriers are particularly concerning given the substantial costs and poor outcomes associated with delirium superimposed on dementia, underscoring the need for scalable, nonintrusive methods capable of identifying delirium-related episodes in home and care home settings.

In recent years, digital health technologies and passive monitoring have shown promise for identifying early warning signs of clinical deterioration in dementia populations. Smart home environments equipped with multimodal sensors offer a particularly compelling approach for delirium-related anomaly detection in people with dementia [12]. These systems can continuously capture life activities and passive physiological measures, dramatically reducing the burden of manual assessment while enabling real-time, individualized monitoring. Previous research has demonstrated that smart home data can successfully detect clinically relevant conditions in dementia populations, including agitation [12] and urinary tract infections (UTIs) [13]—a known precipitant of delirium [14]. Despite this progress, the detection of delirium-related anomalies specifically in home settings remains largely unexplored. Existing work has either applied measurement tools to detect delirium at home [15,16] or used machine learning to detect it in intensive care units and other clinical settings [17,18], leaving a critical gap in the development of passive, home-based approaches for the dementia population. This study aims to address this gap.

The Technology Integrated Health Management (TIHM) project [19] dataset contains continuous behavioral and physiological data collected from people living with dementia using unobtrusive smart home devices over extended periods, providing a unique opportunity to characterize individualized, within-person deviations across delirium-relevant markers and to flag potential delirium risk states in naturalistic home settings. Building on this opportunity, this study introduces a proof-of-concept framework for detecting early delirium-related changes in dementia using theory-driven digital proxies aligned with CAM symptom domains, including agitation, disorientation, and sleep-wake cycle disturbance, alongside additional clinically relevant indicators, such as early warning scores and UTIs. We developed individualized anomaly detection models trained on passively collected longitudinal data to evaluate the feasibility of early delirium risk recognition outside acute care settings. In the absence of clinically validated delirium labels, we deliberately avoided supervised diagnostic modelling and instead adopted an unsupervised, CAM-grounded approach, interpreting detected anomalies as hypothesis-generating, delirium-consistent events rather than diagnostic predictions.

## Methods

### TIHM Dataset

This study used the TIHM dataset [19], and the participants were recruited from Old Age Psychiatry services and were eligible if they were aged over 50 years, had a verified diagnosis of dementia or mild cognitive impairment, and had the capacity to provide informed consent. Each participant was also required to have a study partner or caregiver who had known them for at least 6 months and could attend research assessments. Individuals with unstable mental health symptoms or terminal illness were excluded. This dataset comprises multimodal sensor data from 56 people living with dementia (aged 70-100 years) monitored over an average of 50 days per participant (2803 total person-days). Of these, 13 participants were included in the analysis because they had valid sliding windows, as described in the Data Analysis for Delirium-Related Anomaly Detection section. Continuous data were collected via passive infrared (PIR) motion sensors, door contact sensors, thermometer, and undermattress sleep mats, providing high temporal resolution for activity patterns, body/skin temperature, and minute-by-minute sleep monitoring (sleep states, respiratory rate, and snoring state). The PIR sensors register ambient motion within a room and do not attribute movement to a specific occupant; recorded activity may therefore include caregiver as well as participant movement. Daily physiological measurements included blood pressure, heart rate, weight, and total body water collected through Bluetooth-enabled devices operated manually by participants or caregivers. The dataset includes validated labels for 6 health-related events (agitation, abnormal blood pressure, abnormal temperature, dehydration, abnormal heart rate, and weight changes) confirmed by their monitoring team. This dataset has previously been used to develop models for detecting agitation and UTIs in dementia populations [12,13].

### Feature Engineering

#### CAM-Aligned Markers

##### Agitation

The CAM criteria, descriptions/examples, and proxy features used in this study are presented in Table 1. The agitation labels were provided in the dataset, and they were obtained through a 2-stage verification process involving both automated detection (the details can be seen in the study by Enshaeifar et al [12]) and clinical validation. First, the system generated initial alerts using automated detection, which flagged potential agitation events by identifying outlying values in the continuously collected sensor data. These automated alerts were then forwarded to a clinical monitoring team consisting of health care practitioners who monitored the data around-the-clock. The clinical team validated each alert by directly contacting their caregiver to confirm whether the flagged event was truly an episode of agitation or a false positive. The validated labels were then updated in the database.

**Table 1:** Confusion Assessment Method (CAM) criteria, descriptions or examples, and proxy features used in this study.

CAM items	Item descriptions or examples	Proxy features
Acute onset and fluctuating course	An acute change in mental status	—^a^
Inattention	Such as being easily distractible, or having difficulty keeping track of what was being said	—
Disorganized thinking	Such as rambling or irrelevant conversation, unclear or illogical flow of ideas, or unpredictable switching from subject to subject	—
Altered level of consciousness	Overall level of consciousness	—
Disorientation	Misidentifying the place or time of day	Activity entropy
Memory impairment	Memory problems	—
Perceptual disturbances	Such as hallucinations, illusions, or misinterpretations	—
Psychomotor agitation	Such as restlessness, tapping fingers, or making frequent, sudden changes of position	Agitation
Psychomotor retardation	Such as sluggishness, staying in one position for a long time, or moving very slowly	—
Altered sleep-wake cycle	Such as excessive daytime sleepiness with insomnia at night	Sleep quality score

^a^No proxy feature corresponded to the CAM item.

##### Altered Sleep-Wake Cycle by Sleep Quality Score

This marker is defined as the disturbance of the sleep-wake cycle, such as excessive daytime sleepiness with insomnia at night. We approached this measure by calculating the sleep quality score, computed as the mean of the scores assigned to

each sleep bout during the night, based on the scoring algorithm defined in a previous study [13]. Specifically, based on the duration of each sleep bout, we assigned a score of 2 to bouts with durations over 45 minutes, a score of 1 to bouts with durations between 25 and 45 minutes, and a score of 0 to bouts with durations of fewer than 25 minutes. These thresholds are not based on established clinical or empirical standards; rather, they were found to be useful in that study for categorizing sleep durations. The scores were averaged daily for all bouts during nighttime (10 PM-6 AM).

##### Disorientation by Activity Entropy

Although disorientation, a key symptom of delirium, is difficult to capture directly with the current dataset, we approached it by calculating the entropy rate of location transitions. The “entropy rate” quantifies the randomness of an individual's patterns in their day-to-day life and was used to identify unusual patterns in a previous study [13]. The procedures for obtaining the entropy rate for each individual were detailed in the previous study using a Markov chain model [13], and the major steps can be described as follows: first, we transformed the activity data into a transition matrix, where rows represent the previous location, columns represent the next location, and the values indicate the counts of transitions from one location to another. The entropy rate for each day’s transition matrix was calculated using the formula: H(X) = −^P^*_ij_ π*_ı_*p_ij_logp_ij_*, where H(X) is the entropy rate, *π*_ı_ is the stationary probability of state i, and ^P^*_ij_* is the transition probability from state i to state j. The entropy rate serves as an index of the unpredictability of transitions, with higher entropy values indicating less predictable or more chaotic spatial patterns.

**Figure 1:**
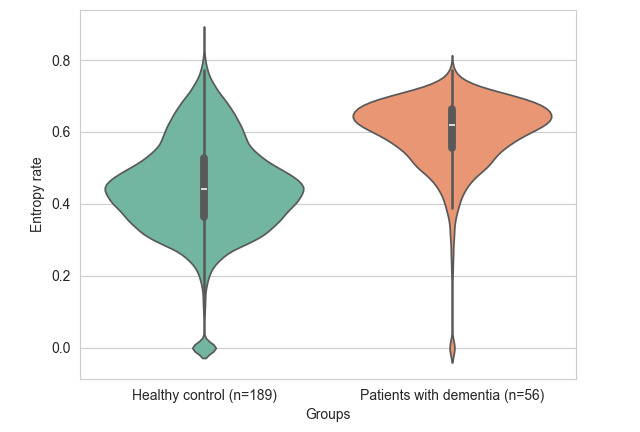
Violin density plot of individual entropy rates of location transitions in the CASAS (Center for Advanced Studies in Adaptive Systems) healthy control sample and in the TIHM (Technology Integrated Health Management) sample of patients with dementia. This CASAS dataset contains longitudinal ambient data collected from 189 community homes over 18 years, from 2007 to 2024. The activity data format in CASAS is very similar to that in TIHM, with timestamps and corresponding locations, so the calculation procedures are identical. To compare the entropy rates between TIHM patients and the healthy control group in the CASAS, we normalized the individual entropy rates in both datasets by the logarithm of the number of locations.

Additionally, exploratory analyses suggest that our method, which uses disorientation approximated by activity entropy rate, detects meaningful patterns related to dementia, although further evidence is needed to establish a direct link to delirium. Specifically, we compared activity patterns from another open dataset, CASAS (Center for Advanced Studies in Adaptive Systems) [20], which includes data from a healthy control group. Our analysis revealed that the median entropy rate for dementia patients was approximately 0.2 higher than that of the healthy controls (Figure 1), providing indirect support that activity entropy could serve as a useful indicator of dementia-related cognitive deterioration and potentially delirium-related changes.

#### Additional Delirium-Relevant Markers

##### Early Warning Score

Early warning scores are recommended as part of the early recognition and response to patient deterioration, demonstrating a strong ability to discriminate patients at risk of adverse outcomes based on routine vital sign measurements [21]. Since delirium is often precipitated by acute physiological disturbances [22,23], incorporating early warning scores as a marker enables the detection of physiological instability that commonly underlies or accompanies delirium-related episodes. Therefore, the early warning score was calculated to summarize individuals’ physiological parameters, following the formula from a previous study [13], as presented in Table 2.

**Table 2:** Description of early warning score calculation.

Physiological parameters	Score						
	3	2	1	0	1	2	3
Systolic blood pressure (mm Hg)	≤90	91-100	101-110	111-219	—^a^	—	≥220
Pulse (per minute)	≤40	—	41-50	51-90	91-110	111-130	≥131
Temperature (°C)	≤35	—	35.1-36	36.1-38	38.1-39	≥39.1	—

^a^Scores are repeated to show that abnormal values on either side of the normal range may receive the same score. Em dashes indicate that no value range applies.

##### Urinary Tract Infection

UTI has been found to be one of the leading precipitating factors for delirium in community-dwelling older adults [23], and a systematic review has identified evidence supporting an association between delirium and UTI [24]. Therefore, UTI is highly informative for delirium-related anomaly detection in dementia patients. UTI cases in this study were operationally identified when a participant exhibited a body temperature outside the range of 36 °C to 38 °C in conjunction with more than 10 bathroom visits within a single day.

### Data Analysis for Delirium-Related Anomaly Detection

Data were segmented into sliding windows comprising 10 baseline days and an 11th test day. This sliding window length was selected to construct each participant’s individual behavioral baseline, consistent with prior work showing that 7- to 14-day windows are effective for modelling recent behavioral patterns [25]. Across the 13 participants, the median proportion of missing calendar days within each individual’s recording period was 2.6% (range 0.0%-31.6%). Missing data were handled using a 2-step procedure. A window was deemed invalid and excluded if the test day contained missing data or if at least 5 baseline days (50% of the baseline window) were missing. Overall, a median of 20.5% of candidate windows were discarded (range 3.4%-97.1%). Second, for the remaining valid windows, missing values for each predictor were imputed using the within-window median for that predictor for the same individual. The 2-step rule and the 50% threshold were adopted from previous research on handling missing data in digital health technologies [26,27].

The Isolation Forest and Long Short-Term Memory (LSTM) models were selected for delirium-related anomaly detection because both are suitable for multivariate time-series data. Anomalies detected by either algorithm were treated as proxies for delirium-related events. All markers derived above were used as features for delirium-related anomaly detection within each individual, including daily agitation count, daily sleep quality scores, daily activity entropy rate, daily early warning scores, and daily UTI occurrence. Both models used identical feature sets and preprocessing procedures. The primary analysis used the Isolation Forest algorithm implemented using the scikit-learn library in Python [28], with 20 estimators, a sample size of 100% of the total sample, and a contamination rate of 10%. For each window, the model was trained on the preceding 10 days of data and applied to the subsequent day, which was classified as anomalous or nonanomalous. This rolling-window approach allowed the Isolation Forest to capture recent data distributions, yielding time-sensitive and adaptive predictions relative to each individual’s baseline.

To quantify the contribution of individual features to anomaly detection, a permutation-based importance analysis was conducted for anomaly events. Within each sliding window, values of a single feature were permuted 20 times while holding all other features constant. The importance of that feature was defined as the difference between the average anomaly score of the 11th day obtained from the permuted data and the corresponding score from the original data. This procedure was repeated for each feature within an anomaly event and subsequently across all anomaly events for a given individual. Larger permutation importance scores indicate a greater contribution of the corresponding feature to the detected anomaly.

**Figure 2:**
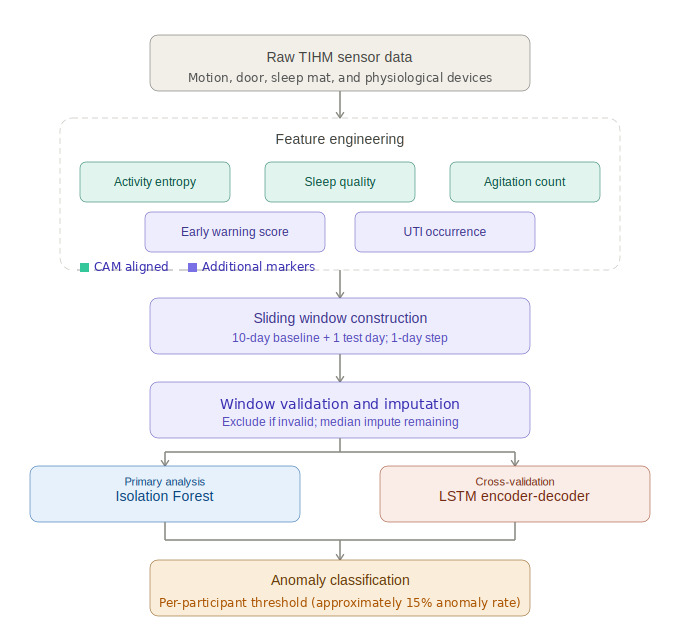
Workflow of delirium-related anomaly detection. CAM: Confusion Assessment Method; LSTM: Long Short-Term Memory; TIHM: Technology Integrated Health Management; UTI: urinary tract infection.

As a cross-methodological comparison, we additionally conducted an analysis using an LSTM model [29]. The features were min-max scaled to the (0, 1) within individuals for the LSTM. The LSTM was implemented as a sequence-to-sequence autoencoder comprising an encoder LSTM layer (32 units) whose final hidden and cell states were passed through a RepeatVector of length 10 to a decoder LSTM layer (32 units, return sequences enabled), followed by a TimeDistributed dense output layer with 5 units to reconstruct the input sequence. One model was trained per participant on that participant’s baseline windows for 20 epochs with a batch size of 4, using the Adam optimizer (default learning rate of 1e-3, no scheduler) and mean squared error as the reconstruction objective. Specifically, for each participant, one LSTM encoder-decoder model was trained on all available baseline windows pooled together, learning to reconstruct normal 10-day sequences for that individual. At scoring time, the trained individualized model reconstructed both the 10-day baseline sequence and the test-day data within each sliding window, yielding a baseline and a test reconstruction error, respectively. The anomaly score was defined as the difference between these 2 errors. Thus, each test day was evaluated relative to its own preceding 10-day baseline, preserving window-level temporal context at inference while being more computationally efficient than training a separate model for each window. The anomaly quantile for the LSTM was set at 15% to match that of the Isolation Forest. The agreement rate between the two methods was calculated. The data analysis workflow is presented in Figure 2.

### Ethical Considerations

The TIHM study received ethics approval from the London-Surrey Borders Research Ethics Committee (TIHM 1.5 REC: 19/LO/0102), and the dataset has been made public with participants fully anonymized. Participants provided informed consent prior to participation. Each participant was required to have a study partner or caregiver who had known them for at least 6 months and was able to attend research assessments. Where participants were unable to reliably report information regarding their health, the study partner or caregiver completed the relevant assessments on their behalf. All the participants have granted the publication of this dataset. More details can be retrieved from the original paper [19].

## Results

In total, 13 patients were included in the anomaly detection analysis. These patients were monitored for a median of 56 (range 18-83) days, yielding a total of 735 person-days of observation. All detected delirium-related events are represented as red dots for each marker and can be accessed via the open-source materials of this study [30].

**Figure 3:**
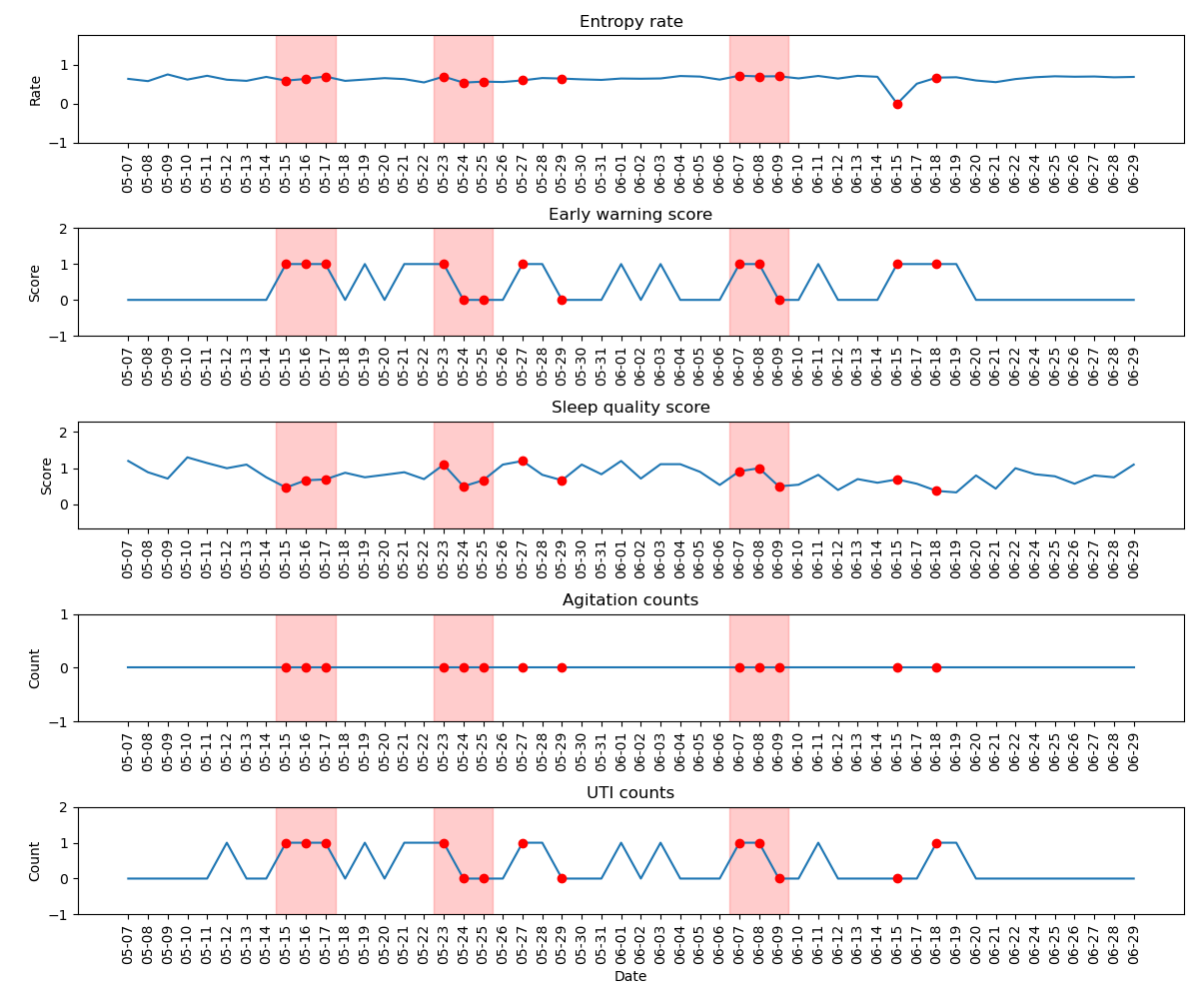
Representative anomaly detection results (red dots) for one patient (pseudo IDs=30a32) using the multivariate Isolation Forest algorithm. Anomaly clusters were highlighted with red shading. UTI: urinary tract infection.

Future validation studies with labeled data are needed to optimize threshold selection for each method. For comparison, we used an anomaly threshold of approximately 15% across all individuals for both models, which enabled comparison independent of threshold stringency. For the Isolation Forest, the median proportion of anomalous days was 11.11% (range 0%-30.77%), with anomalies typically occurring in short temporal clusters rather than as isolated events. One example of these temporal clustering patterns is shown in Figure 3. Notably, the agreement rates between Isolation Forest and LSTM ranged from 0% to 40% across individuals. Agreement tended to be higher when both algorithms detected more anomalies. Details are presented in Table 3.

**Table 3:** Agreement in detected anomaly events between the two algorithms.

Participant ID	Total days^a^	Shared days^b^	IF^c^-only days^d^	LSTM^e^-only days^c^	Agreement rate (%)^f^	IF clusters, n^g^	LSTM clusters, n^g^	Shared clusters, n^g^
16f4b	2	0	0	1	0	0	0	0
1fbe4	39	4	8	2	28.6	4	1	1
30a32	52	5	8	3	31.2	3	1	1
55cd4	50	6	7	2	40	1	2	1
93c14	27	1	2	3	16.7	0	0	0
96adf	39	3	3	3	33.3	1	1	1
a2849	47	2	5	5	16.7	1	1	0
c55f8	71	3	7	8	16.7	1	1	0
c5785	56	1	5	8	7.1	0	1	0
c8574	35	1	0	5	16.7	0	0	0
d7a46	1	0	0	0	0	0	0	0
e2472	18	0	1	3	0	0	0	0
ec812	55	3	2	6	27.3	0	2	0

^a^Total days refers to the total number of test days for each participant, equivalent to the total number of sliding windows available for that individual.

^b^Shared days refer to the number of anomaly days detected by both algorithms.

^c^IF-only days and LSTM-only days refer to anomaly days detected only by the Isolation Forest and Long Short-Term Memory algorithms, respectively.

^d^IF: isolation forest.

^e^LSTM: Long Short-Term Memory.

^f^The agreement rate was calculated as the number of shared anomaly days divided by the union of all anomaly days detected by either algorithm.

^g^An anomaly cluster was defined as a sequence of 2 or more consecutive calendar days with detected anomalies; the numbers of IF-only, LSTM-only, and shared clusters are also reported here.

**Figure 4:**
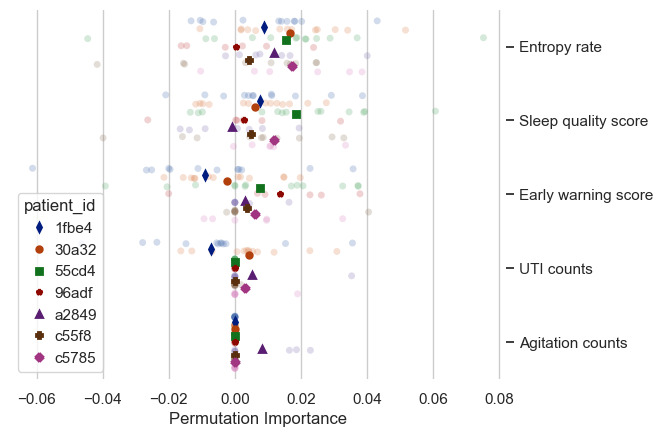
Feature permutation importance across anomaly events and individuals. Individual points represent the permutation-induced change in anomaly score, defined as the difference between the observed anomaly score for an identified event and the score obtained after feature permutation. Markers indicate the mean permutation importance across anomaly events within each individual for each feature; marker shape and color distinguish individuals. Larger importance scores indicate a greater contribution of the corresponding feature to anomaly detection. To improve estimate stability, only individuals (n=7) with more than 5 anomaly events identified by the Isolation Forest algorithm were included in this visualization. UTI: urinary tract infection.

**Figure 5:**
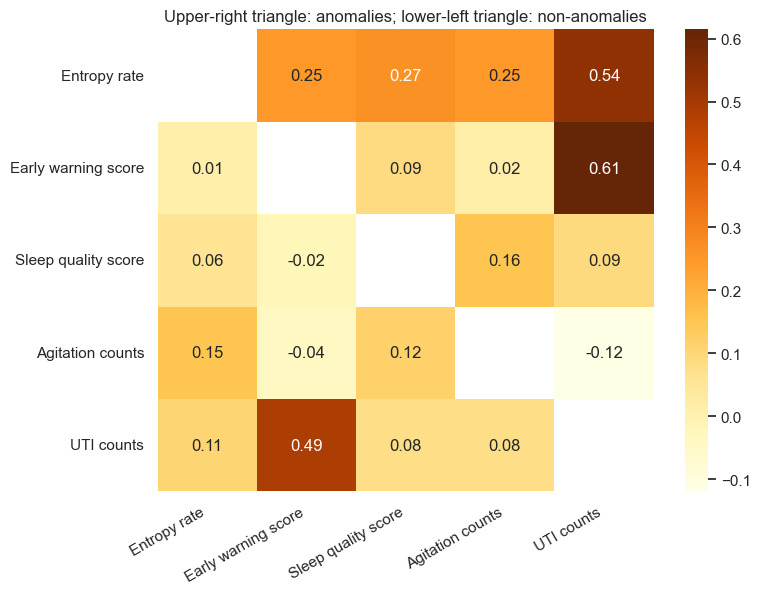
Spearman correlation strength between features for nonanomalous and anomalous events. The lower-left triangle shows correlations computed from events classified as nonanomalies, whereas the upper-right triangle shows correlations computed from events classified as anomalies. UTI: urinary tract infection.

For the primary Isolation Forest analysis, we also examined the patterns of feature importance across anomaly events. Activity entropy, sleep quality scores, and early warning scores were the most influential features, though their relative importance varied substantially across individuals (Figure 4). To improve estimate stability, only individuals (n=7) with more than 5 anomaly events identified by the Isolation Forest algorithm were included in this visualization. Case example in Figure 3 illustrates how acute deviations across multiple CAM-aligned markers co-occurred during anomaly periods, while Figure 5 further analyzes the Spearman correlation strength between features during anomaly versus nonanomaly events. The stronger interfeature correlations observed during anomaly events support the presence of co-occurring deviations among features associated with delirium-related episodes.

## Discussion

### Principal Findings

This study demonstrates the feasibility of using theory-driven markers to detect delirium-related patterns in home settings for individuals with dementia, addressing the challenges of clinical delirium assessment, particularly in dementia populations. By applying unsupervised anomaly detection algorithms to within-individual data using sliding windows, this approach ensures the time sensitivity of detection while accounting for individual differences in symptom deviations. Although validated labels for delirium episodes were not available, the detection of multivariate anomalies in home sensor data using markers aligned with CAM criteria provides confidence that these anomalies correspond to known delirium symptoms. Furthermore, several additional findings discussed below further support the validity of this detection approach.

First, we observed that numerous detected delirium-related episodes show patterns of temporal clustering, which align with known delirium patterns [31]. Second, we observed that for some clustered delirium-related episodes detected, the composing markers demonstrate concurrent deteriorations. This also aligns with previous findings that the mean number of CAM symptoms preceding delirium episodes is 2.4, and the mean number is 3 after episodes [32]. Our result patterns match patterns normally observed in delirium episodes in older adult residents, which provides some evidence for the effectiveness of detection.

We observed variation in agreement rates across individuals between the two anomaly detection algorithms, which reflects methodological differences rather than limitations in the anomaly detection process itself. LSTM models, with their focus on sequential data, are designed to capture longitudinal changes and temporal dependencies in the data [33]. As a result, if the previous data shows more fluctuation, the LSTM model may be more tolerant of deviations, since it inherently learns the patterns of variability over time. In contrast, the Isolation Forest algorithm focuses on the global distribution and deviations of markers within fixed time windows, without considering temporal sequence [34]. As a result, it is more sensitive to extreme deviations from the overall baseline. Given that this study is a proof of concept with flexible modular algorithms for anomaly detection, future datasets with validated labels will be necessary to determine which algorithm is best suited for delirium-related anomaly detection.

### Limitations

However, there are important limitations in this study. First and most importantly, the lack of ground truth labels makes it impossible to determine the accuracy and other performance measures such as sensitivity and specificity of the detection algorithms. While this study provides compelling evidence that the detected delirium-related patterns align with existing literature and that our markers are consistent with CAM criteria, it goes beyond a proof of concept. A key contribution of this study is the introduction of a novel, data-driven approach to delirium-related anomaly detection in home settings, highlighting the potential for continuous, individualized monitoring of delirium-related patterns using theory-driven markers. Second, while we aimed to construct markers that closely align with CAM items, future efforts should focus on collecting more comprehensive data to enable the derivation of additional relevant markers that are unavailable in the current dataset (eg, inattention and altered level of consciousness) and ensure that the proxy markers accurately measure the same constructs as those defined by the CAM items. For instance, circadian disorientation was also included in the disorientation marker [9,35], but this is unavailable in the current dataset. There is a caveat that behavioral proxies such as agitation and entropy cannot fully capture the complexity of cognitive symptoms and therefore do not constitute direct substitutes for clinical cognitive assessment. Rather than replacing individual CAM criteria, our aim is to derive an objective, continuous, and nonintrusive approximation using smart home data, with the goal of achieving detection performance comparable to the CAM.

Apart from the lack of validated labels, the current dataset has several additional limitations that should be addressed in future data collection. First, sleep data were available for only a subset of participants because the mattress sensor was introduced later in the study, and some participants did not consent to its installation. Future translational work should investigate these barriers more directly, particularly whether concerns relate to comfort, privacy, or other acceptability reasons. A further limitation is that the duration of sensor recording varied across participants, resulting in an unequal number of valid baseline–test window pairs per individual. Participants with shorter recording periods contributed fewer windows, which may have reduced both the opportunity to detect anomalies and the stability of the personalized behavioral baseline. Although the 10-day sliding window approach does not in principle require very long longitudinal data, longer monitoring periods would allow a more representative characterization of typical behavior and make the anomaly thresholds more meaningful. These differences in recording duration partly arose from the rolling recruitment design of the ongoing study, which could be improved in future study design, and partly from participant dropout or death, which is an inherent challenge in longitudinal research involving older adults living with dementia.

In addition, several factors may influence how sensor-derived activity patterns are interpreted. The PIR sensors capture motion as discrete events and do not attribute movement to a specific individual, so caregiver presence may contribute to recorded activity. This concern is mitigated in 2 ways. First, because anomalies are defined as deviations from each participant’s own preceding baseline, habitual caregiver activity is incorporated into that baseline and does not by itself generate anomalies. Second, of the 5 markers, only activity entropy is derived purely from ambient motion; sleep quality, early warning score, UTI, and agitation are substantially patient specific, and our feature importance analysis (Figure 4) indicates that detection is driven by a combination of these markers rather than by entropy alone. Nevertheless, because the open TIHM dataset does not record living arrangements or the proportion of time a participant is unaccompanied, we could not stratify analyses by co-residence or occupancy, and contextual influences cannot be fully ruled out. We recommend that future data collection enable patient-specific measurement of activity, for example, through patient-specific PIR sensing, to ensure that recorded signals correspond only to the target patient.

### Contributions

Nevertheless, this study contributes to detecting delirium-related anomalies in home settings in several important aspects. First, this is the first study to work on detecting delirium-related anomalies in smart home settings and provides evidence of viability, which has huge potential for prevention and early intervention for people living with dementia and reducing costs for hospital admissions. Second, our individualized, unsupervised detection framework is well aligned with real-world implementation contexts, in which delirium-related episodes are difficult to clinically validate in a timely manner. By modelling within-person baselines rather than relying on global population models, this approach accommodates substantial interindividual heterogeneity and captures dynamic shifts in an individual’s baseline as biological states and daily behaviors evolve over time. This study lays a strong foundation for future work, including developing data collection protocols that better align with the goal of delirium-related anomaly detection, and algorithm improvement and validation through clinical partnerships.

### Conclusions

This proof-of-concept study demonstrates the feasibility and promise of using smart home data to detect anomalies consistent with delirium superimposed on dementia, with potential value in reducing health care costs through early intervention. The limitations of lacking clinically confirmed labels and the strengths of detecting anomalies while considering individual differences are acknowledged. Future work requires data collection with a focus on delirium prevention, including data that allow features better aligned with delirium symptoms, as well as clinically confirmed labels for ground truth validation.

## Data Availability

The datasets generated or analyzed during this study are available in the corresponding Zenodo repository [20].

## Abbreviations

CAM: Confusion Assessment Method
CASAS: Center for Advanced Studies in Adaptive Systems
LSTM: Long Short-Term Memory
PIR: passive infrared
TIHM: Technology Integrated Health Management
UTI: urinary tract infection
